# Association between body mass index and treatment completion in extended-release naltrexone-treated patients with opioid dependence

**DOI:** 10.3389/fpsyt.2023.1247961

**Published:** 2023-08-02

**Authors:** Xinyi Li, Daniel D. Langleben, Kevin G. Lynch, Gene-Jack Wang, Igor Elman, Corinde E. Wiers, Zhenhao Shi

**Affiliations:** ^1^Department of Psychiatry, University of Pennsylvania Perelman School of Medicine, Philadelphia, PA, United States; ^2^National Institute on Alcohol Abuse and Alcoholism, Bethesda, MD, United States; ^3^Department of Psychiatry, Cambridge Health Alliance, Harvard Medical School, Boston, MA, United States

**Keywords:** opioid use disorder, medication-assisted treatment, body weight gain, addiction, food, obesity, eating

## Abstract

**Background:**

Excessive consumption of opioids is associated with impaired metabolic function including increased body mass index (BMI). Opioid antagonist naltrexone (NTX) is an effective treatment for opioid use disorder (OUD) that has the potential to mitigate such metabolic disturbances. Understanding the relationship between treatment adherence and BMI in NTX-treated OUD patients may provide valuable insights into optimizing clinical outcomes.

**Methods:**

Patients with opioid dependence were offered up to three monthly injections of extended-release (XR) NTX. Treatment completers (n = 41) were defined as those who had received all three XR-NTX injections, and non-completers (n = 20) as those missing at least one injection. Logistic regression was performed to examine the association between pre-treatment BMI and treatment completion.

**Results:**

BMI was positively associated with treatment completion. This association remained significant after adjusting for potentially confounding variables.

**Conclusion:**

Our findings suggest that baseline BMI may serve as a potential predictor of XR-NTX treatment adherence in patients with OUD and could help healthcare providers and policy makers alike in developing strategies to improve retention and tailor interventions for specific patient subgroups.

## 1. Introduction

Opioid use disorder (OUD) is a serious global public health issue affecting over 16 million individuals worldwide (1). In the United States, OUD ranks as one of the leading causes of disability-adjusted life-years (2–5). OUD is associated with excessive body weight gain (BWG) leading to overweight and obesity (6–8). Exogenous opioids enhance μ-opioidergic neurotransmission (9, 10) that not only stimulates neuropeptides that promote appetite but also inhibits neuropeptides that suppress appetite (11). The enhanced μ-opioid neurotransmission further modulates eating behaviors by increasing the preference for pleasurable and indulgent foods high in sweetness and fat (12, 13). Furthermore, chronic exposure to opioids and substances of abuse produces neuroadaptations that promotes overeating during abstinence due to the lack of the reinforcing effects of the substances (14). As a result, these mechanisms (11) may play a role in the development of food cravings and even food addiction (15–18).

Besides negative psychosocial impacts (low self-esteem and societal stigmatization) of BWG (19), OUD patients are particularly susceptible to the detrimental medical sequelae of BWG such as the Metabolic Syndrome (20), which is a cluster of cardiovascular risk factors, including abdominal adiposity, insulin resistance, impaired glucose tolerance, dyslipidemia, and hypertension (21). These effects may contribute to the high rates (22, 23) of treatment non-adherence with the OUD medication-assisted treatment (MAT) involving opioid agonists such as buprenorphine and methadone (24, 25), which, despite their positive clinical outcomes in terms of reducing overdose mortality, infectious diseases, crime, and societal impact (26, 27), also elicit concerns about their potential negative effects on eating habits and consequent unwanted BWG (20, 28, 29). In contrast, the remaining MAT constituent (30), opioid antagonist naltrexone (NTX), has been associated with diminished sweet taste’s hedonic appeal (31), along with decrements in food intake, body fat mass (32) and BWG (33). Moreover, in combination with bupropion, NTX is commonly prescribed for weight management in individuals who are overweight or obese (34). Likewise, NTX analog, samidorphan (35), counteracts BWG arising in the context of antipsychotic therapy (36). Heightened BMI can be associated with negative affective states, body image concerns or weight-related stigmatization that may impact patients’ self-esteem or self-efficacy driving their engagement with treatment (19). The anticipated alleviation of opioid-induced metabolic effects through the opioid receptor blockade by NTX could potentially enhance adherence to NTX treatment through negative reinforcement (11, 37).

The assessment of adherence to medication often relies on self-reports and collateral information (38) as well as pill counts (39), electronic monitoring devices (40–42), biomarkers or blood tests (43), direct observation of intake (44), pharmacy records (45), mobile apps and digital tools (46). Assessing oral NTX adherence in OUD patients remains a daunting task (42) due to conscious and unconscious denial, cognitive deficits secondary to the use of mind-altering substances, fear of disclosure and lack of trust (47) in the healthcare system and in the clinical research establishment (25, 48, 49). The monthly injectable, extended-release NTX (XR-NTX) is associated with better adherence than oral NTX, and such adherence can be easily assessed objectively. Nevertheless, adherence to XR-NTX varies across individuals, and there is a particularly high rate of premature dropout during the first 3 months of treatment (50–52). It is challenging because relapse to opioids almost invariably follows non-adherence to XR-NTX, and repeat detoxification is required before resuming XR-NTX (53, 54). Relapse after discontinuing XR-NTX also poses a high risk of fatal overdose due to the loss of tolerance to opioids (55) and hypersensitivity of opioid receptors (56). Efforts to improve adherence have identified incentives for continual treatment, such as physicians and businessmen with professional and financial reasons to remain abstinent (57), and neurobiological markers (54, 58). However, the effectiveness of these approaches in improving treatment adherence remains unclear.

To that end, we operationalized adherence as receipt of all three XR-NTX injections offered on the present study (i.e., treatment completion), while non-completers were those who have missed at least one injection (37, 52, 58). While this is just one possible definition of treatment adherence (59), we have chosen such approach because it provides a clear, measurable, and unambiguous definition. In addition, it is not affected by other variables that may be subjected to missing data (e.g., urine toxicology screenings), ensuring a more accurate estimate of the variable of interest. Building on the above considerations, we hypothesized that higher BMI would be associated with more treatment adherence to XR-NTX in opioid dependent patients.

## 2. Methods and materials

### 2.1. Participants

The study is a retrospective, secondary analysis of data collected by two intervention studies in 61 detoxified OUD patients (37, 58, 60). Participants of both studies were offered free, medically supervised, 3-month treatment with XR-NTX for OUD. One study stipulated intravenous heroin as the drug of choice (37), whereas the other included both individuals who use heroin and prescription pill (58, 60). Inclusion criteria were (1) DSM-IV-TR diagnosis of opioid dependence, established using the best estimate format based on all available information (including history and physical examination and the Mini-International Neuropsychiatric Interview) (2), active opioid use for more than 2 weeks in the 3 months prior to detoxification, and (3) good physical health, as evidenced by clinical examination. Participants were excluded if they demonstrated (1) any current major psychiatric disorder (i.e., psychosis, dementia, and schizophrenia) (2), current major medical problems, head trauma, or neurological disorder (3), current use of potentially confounding medications, including anti-dopaminergic agents, anticonvulsants, and beta-blockers, or (4) being pregnant or breastfeeding. Detailed information on the studies was reported elsewhere (37, 58, 60).

### 2.2. Study procedures

Participants’ weight and height and demographics were assessed at screening prior to treatment. Weight was measured using a digital electronic scale and height with a Harpenden stadiometer, calibrated on a weekly basis. Opioid craving was scored subjectively using a 10-point scale (0 = none; 9 = extremely) (60), for which data were unavailable for three participants.

Following complete opioid detoxification, as confirmed with a negative naloxone challenge test, subjects were offered up to three monthly intramuscular injections of XR-NTX (380 mg extended-release naltrexone-HCl, Vivitrol®, Alkermes Inc., Cambridge, MA). Sixty-one individuals having received at least one XR-NTX injection and for which baseline BMI measurements were available were included in the analysis. Treatment completers were categorized as individuals who have received all three monthly injections of XR-NTX, whereas non-completers only received one or two of the injections.

### 2.3. Data analysis

All statistical analyzes were performed with R.^1^ The association between treatment completion (0 = non-completion, 1 = completion) and BMI was examined by logistic regression. Due to the observational design of the study and the lack of randomization, we investigated the effects of controlling for other baseline variables (see Table 1) using the purposeful variable selection procedure (61). Specifically, each variable was tested individually for its association with treatment completion using a simple logistic regression. Variables that reached *p* < 0.25 were selected as candidates and entered in a multivariable logistic regression that tested their joint association with treatment completion. If the variable that had the largest value of p in the multivariable model reached *p* < 0.10, or if removing it from the model resulted in a change in any remaining parameter estimate greater than 15%, then the variable was retained in the model, and the variable that had the second largest value of p was subjected to the same examination. Otherwise, the variable with the largest value of p was removed, and a reduced multivariable logistic regression model was examined. The process was iterated until all remaining variables in the model were determined eligible to be retained. Lastly, the non-candidate variables that were not selected for the initial multivariable model were included one at a time, starting with the one that had the smallest value of p in the simple logistic regression analysis. The non-candidates that reached *p* < 0.15 in the multivariable model or caused a change in any remaining parameter estimate greater than 15% were retained in the model. Given that BMI was the variable of interest, and that data were obtained from two different studies, we included BMI and study (Study 1 vs. Study 2) in all multivariable models regardless of their *p*-values and effects on the coefficient estimates of other variables. The variable race was dichotomized to Caucasian vs. non-Caucasian because of the small number of individuals of other races. Due to the limitations of the Wald test in small and unbalanced samples (62, 63), we used the likelihood-ratio test to evaluate coefficient significance. Group data were summarized as mean ± standard deviation (SD). All analyzes were two-tailed, and *p* < 0.05 defined statistical significance.

**Table 1:** Participant characteristics (mean ± SD or number of occurrences).

Variable	Non-completer	Completer	*P*-value^1^
*N*	20; 5 (Study 1), 15 (Study 2)	41; 16 (Study1), 25 (Study 2)	0.27
Age (year)	26.5 ± 6.9	30.2 ± 9.4	0.10
Sex	10 female, 10 male	15 female, 26 male	0.32
Race	18 Caucasian, 1 AA, 1 Asian	36 Caucasian, 4 AA, 1 Asian	0.61
Education (year)	13.3 ± 2.3	13.9 ± 2.0	0.28
BMI (kg/m^2^)	22.7 ± 1.6	25.8 ± 4.4	0.002
Preferred opioid	12 heroin, 8 prescription	24 heroin, 17 prescription	0.91
Opioid craving (range: 0–9)	4.3 ± 2.6	3.0 ± 2.6	0.08

AA, African American; BMI, body mass index.

1 Simple logistic regression analysis.

The following two considerations guided our choice of a binary outcome (i.e., completion vs. non-completion). The first consideration was clinical relevance, as completion reflects the extent to which individuals have followed through with the entire *a priori* recommended treatment protocol. The second consideration was pragmatism, as binary categorization is easier to analyze and communicate in clinical settings compared to the varying number of injections.

## 3. Results

A total of 61 participants received at least one dose of XR-NTX, of whom 41 were completers and 20 were non-completers (see Methods). Baseline characteristics of included participants are presented in Table 1. BMI ranged from 18.9–36.2, consisting of normal weight (*n* = 40), overweight (BMI > 25; *n* = 12) and obese (BMI > 30; *n* = 9). Remarkably, 91% (*n* = 19 out of 21) of participants in the overweight/obese category vs. 55% (*n* = 22 out of 40) of participants with a normal weight completed the study treatment with three monthly XR-NTX injections (*p* < 0.01, Fisher Exact Test).

**Figure 1:**
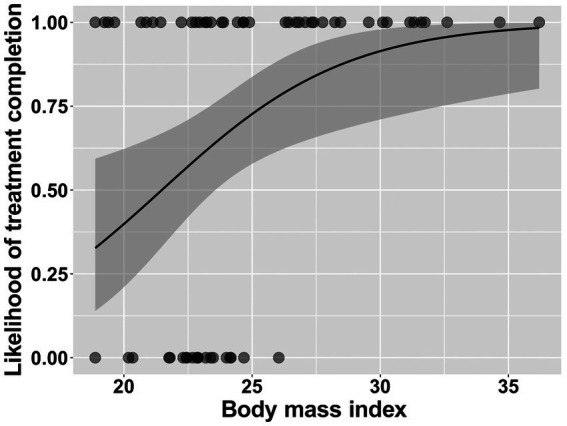
Simple logistic regression model of treatment completion predicted by body mass index. The shaded area represents 95% confidence interval.

Logistic regression model showed that higher BMI was significantly associated with higher likelihood of treatment completion (B = 0.28, SE = 0.10, *p* = 0.002; exp.(B) = 1.32, 95% confidence interval = [1.10, 1.67]; *χ*^2^(1) = 10.01, Nagelkerke *R*^2^ = 0.21; see Figure 1). The association between treatment completion and each of the other baseline variables is summarized in Table 1.

During the purposeful model selection procedure, craving and age were selected as candidates for multivariable logistic regression, in addition to BMI and study (Study 1 vs. Study 2). Age was subsequently eliminated from the multivariable model, and education was added back. BMI remained a significant predictor of treatment completion. The final multivariable model is summarized in Table 2.

**Table 2:** Multivariable logistic regression model of treatment completion.

Variable	B	SE	*P*-value
BMI	0.44	0.15	<0.001
Study (1 & 2)	2.59	1.00	0.002
Opioid craving	−0.34	0.16	0.005
Education (year)	0.29	0.18	0.085
χ^2^(4) = 24.15, *p* < 0.001, Nagelkerke *R*^2^ = 0.47			

SE; standard error; BMI, body mass index.

## 4. Discussion

The present study examined the association between BMI and treatment completion in opioid dependent patients that were offered up to three monthly injections of XR-NTX. The results indicate that higher BMI was associated with a higher likelihood of treatment completion, suggesting that individuals with higher BMI may demonstrate better adherence to XR-NTX therapy. It is reasonable to assume that improved BMI and related metabolic outcomes would strongly motivate continued adherence to XR-NTX.

Nonetheless, a comprehensive range of emotional, motivational, and cognitive processes are involved in planning a recovery path, assessing the risk of relapse or deterioration in opioid consumption, and evaluating the outcomes of ongoing XR-NTX therapy. Changes in any of these mechanisms can determine whether a patient gains control over opioid addiction or whether the addiction gains control over that patient. According to Prospect Theory (64), individuals subjectively evaluate outcomes through elaborate mental processes. These processes involve adjusting their expectations (i.e., prospects) regarding the likelihood of continued overweight/obesity or successful weight loss based on an acceptable level of recovery-related discomfort. This adjustment impacts the coping strategies that patients employ, which in turn influences their expectations and satisfaction with their body weight outcomes. It is possible that protracted withdrawal and/or food craving up to a new neutral state may not be perceived as aversive (65) in such reformulated contextual framing (66, 67), prompting adherence and eventual recovery (37).

On the other hand, the subjective valuation process leads to distinct slopes in the emotional functions of weight gain and loss. The weight gain may typically exhibit a steeper slope than the loss domain (68, 69), contributing to maladaptive coping strategies such as emotional eating, using food for comfort, self-blame, negative self-talk about body image, and engaging in disordered eating behaviors (70, 71). Moreover, since neural circuitries underlying the motivational effects of opioids and palatable food overlap (14, 72, 73) and may sensitize over time, cross-sensitization might occur as well (11, 74). This means that exposure to opioids could increase the consumption of unhealthy food, and vice versa. As such, preclinical evidence suggests that amphetamine-sensitive rats demonstrate cross-sensitization to sugar (75), and that exposure to high-fat diets *in utero* increases later sensitivity to drugs of abuse (14). In short, realization that opioid and food addictions share a common neurobiological foundation (11, 76) has important theoretical and therapeutic implications underscoring the need for biopsychosocial interventions by a multidisciplinary team comprised healthcare professionals, addiction specialists, nutritionists, and mental health providers delivering holistic care and support for individuals navigating weight management concurrently with the treatment of opioid addiction (77).

It is worth noting that therapeutic adherence is a complex entity affected by multiple factors, and BMI alone is unlikely to be a robust predictor in isolation. Individualized assessments and a comprehensive understanding of a patient’s circumstances are crucial for effective treatment planning and adherence support. From the biochemical perspective, higher BMI is associated with greater plasma leptin concentration and food consumption (78, 79). Blood glucose elevations, such as after a meal, trigger the release of insulin along with incretin hormones (e.g., glucagon-like peptide-1 and glucose-dependent insulinotropic polypeptide) that restrain the hedonic/motivational neural pathways otherwise driving the consumption of both palatable food (80, 81) and opioid drugs (11, 20). Moreover, if a patient has a higher BMI and low ratio of lean body mass to fat mass, the pharmacokinetics of naltrexone may be affected, potentially altering the drug’s side effects due to changes in the medication’s volume of distribution, clearance, and metabolism (82).

From social and environmental perspectives, BMI can be linked to many factors (83) that may indirectly influence adherence. For instance, socioeconomic status, access to healthcare, social support networks, and cultural norms related to body weight and medication use can vary among individuals with different BMIs. In fact, some opioid-dependent individuals may even experience weight loss (84, 85) potentially attributable to stress, lifestyle disruptions, such as neglecting nutritional needs or engaging in risky behaviors, which can further contribute to emaciation. Weight loss in opioid dependent patients may indicate malnutrition, poor overall health, and increased vulnerability to complications (84). These factors may impact adherence by affecting medication availability, support systems, or adherence-promoting messages by healthcare professionals. Whatever the case may be, encouraging healthy lifestyle, sleep hygiene, proper access to healthcare, regular exercise and physical activity can not only support weight management but also contribute to improved mood, overall well-being, stress reduction, and therapeutic adherence, which are essential aspects of addiction recovery.

Additionally, our results are consistent with previous findings that craving is associated with poor treatment outcomes in OUD (86–88). The predictive value of craving on drugs seeking behavior extends to other drugs of abuse, including cocaine (89) and methamphetamine (90). Here, we demonstrated a trending effect of baseline craving scores in predicting XR-NTX treatment completion, which became significant after controlling for BMI in the multivariable regression model. Nevertheless, this finding should be interpreted with caution due to the small sample size and utilization of a single-item 10-point subjective craving scale, as opposed to validated questionnaires (91).

It is important to acknowledge the limitations of the study, including its retrospective nature, potential confounders, selection bias, small sample size, and the inclusion of data from two different studies, which may limit the generalizability of the findings. The study also relied on subjective craving scores, which may introduce measurement errors and biases compared to more objective measures of craving and treatment adherence (37, 92, 93). Further research with larger prospective cohorts and other measures of treatment adherence is warranted to validate these findings and explore the underlying mechanisms.

In conclusion, improved BMI and related metabolic outcomes are likely to serve as strong motivators for continued adherence, though the findings need be interpreted with caution given the aforementioned limitations of the study. The association between high BMI and improved therapeutic outcome with XR-NTX, if confirmed by future research, would indicate that XR-NTX should be the default first step if MAT is considered in obese/overweight OUD patients. The findings underscore the importance of the biopsychosocial aspects of opioid addiction treatment and weight management. A multidisciplinary approach can deliver holistic care and support to individuals navigating weight management concurrently with the treatment of opioid addiction. Overall, this study adds to our understanding of the complex interplay between BMI, treatment completion, and adherence in the context of OUD and XR-NTX therapy. By considering the individualized needs and circumstances of patients, healthcare providers can enhance treatment planning and adherence support, leading to improved outcomes in both addiction recovery and optimal weight management.

## Data availability statement

The original contributions presented in the study are included in the article/supplementary materials, further inquiries can be directed to the corresponding authors.

## Ethics statement

The studies involving humans were approved by University of Pennsylvania Institutional Review Board. The studies were conducted in accordance with the local legislation and institutional requirements. The participants provided their written informed consent to participate in this study.

## Author contributions

DL designed the study and collected the data. XL, KL, CW, and ZS analyzed the data. XL, DL, KL, G-JW, IE, CW, and ZS interpreted the results. XL, DL, IE, CW, and ZS wrote the manuscript. All authors provided critical revision of the manuscript for intellectual content and approved the final version of the manuscript.

## Funding

This work was supported by the Commonwealth of Pennsylvania CURE grant SAP#4100055577 (Anna Rose Childress, University of Pennsylvania), the NARSAD Young Investigator Grant from the Brain & Behavior Research Foundation (#30780) (ZS), and the following National Institutes of Health grants: T32DA028874 (XL), R01DA024553 (Charles P. O’Brien, University of Pennsylvania), R00AA026892 (CW), and K01DA051709 (ZS). The funding sources had no role in the study design, collection, analysis, or interpretation of the data, writing the manuscript, or the decision to submit the paper for publication.

## Conflict of interest

The authors declare that the research was conducted in the absence of any commercial or financial relationships that could be construed as a potential conflict of interest.

## Publisher’s note

All claims expressed in this article are solely those of the authors and do not necessarily represent those of their affiliated organizations, or those of the publisher, the editors and the reviewers. Any product that may be evaluated in this article, or claim that may be made by its manufacturer, is not guaranteed or endorsed by the publisher.

## Footnotes

^1^ www.R-project.org
